# Blood biochemistry changes in a minipig infarction model

**DOI:** 10.3389/fvets.2025.1493660

**Published:** 2025-01-27

**Authors:** Dénes Kőrösi, Ágoston Göcző, Noémi Varga, Rita Garamvölgyi, Nándor Balogh, Kornélia Farkas, András Vorobcsuk

**Affiliations:** ^1^Doctoral School in Animal Science, Hungarian University of Agriculture and Life Sciences, Kaposvár, Hungary; ^2^Medical School, University of Pécs, Pécs, Hungary; ^3^Cardio-Econom Ltd., Görcsöny, Hungary; ^4^Praxislab Ltd., Budapest, Hungary; ^5^Medical School, Institute of Bioanalysis, University of Pécs, Pécs, Hungary; ^6^Department of Cardiology, Kaposi Moritz Teaching Hospital, Kaposvár, Hungary

**Keywords:** blood biochemicals, cardiac markers, closed chest infarct, large animal model, minipig, stress

## Abstract

**Introduction:**

The present study aimed to assess changes in biochemical parameters during the adaptation of the myocardial infarction model to a conventional Hungarian minipig breed. According to our hypothesis, changes in the blood level of the necroenzymes are not only related to the interventional procedure but are also influenced by peri-procedural animal keeping and treatment conditions.

**Methods:**

Closed chest acute myocardial infarction (AMI) was induced by balloon occlusion for 90 min in the left anterior descendent coronary artery (LAD) in 24 adult, female Pannon minipigs followed by reperfusion. Blood samples were taken before AMI, and immediately after the reperfusion, during the cardiac magnetic resonance imaging (cMRI) on days 3 and 30. Aspartate transaminase (AST), alanine aminotransferase (ALT), creatine kinase (CK), lactate dehydrogenase (LDH), and high-sensitivity troponin I were determined.

**Results:**

While the parameters measured at baseline remained within physiological ranges, a notable elevation was seen in comparison with the results observed on day 30. This phenomenon was evident in all the laboratory parameters tested, except hs-troponin. The results for AST, ALT, LDH, and CK were statistically significant (*p* = 0.011, *p* = 0.001, *p* = 0.013, and *p* = 0.001, respectively). A statistically significant difference was observed between the baseline and 30-day AST/ALT ratio (*p* = 0.00514).

**Discussion:**

The elevated levels of necroenzymes observed at baseline are likely to be a consequence of the physical and social stress imposed by the study design on the minipigs during the 72-h period prior to intervention. It is essential to define the optimal timing of baseline blood tests to ensure the reliability of the biochemical profile in a large animal infarction model.

## 1 Introduction

Cardiovascular disease (CVD) represents one of the most significant causes of mortality globally, accounting for 31% of all deaths and affecting approximately 17.9 million individuals annually (1). Acute myocardial infarction (AMI) and its associated complication, ischemic heart failure (HF), represent the leading cause of death and permanent disability worldwide. The development of new cardioprotective therapies is required to reduce the size of AMI and prevent HF. This necessitates the performing of translational studies in large animal models. To address this need, pigs represent a suitable alternative to rodents, dogs, and other primates in cardiological research (2, 3). The porcine heart exhibits a striking resemblance to the human heart in its morphological and functional characteristics. This resemblance extends beyond mere anatomy to encompass the heart’s size and perfusion, which are strikingly similar (3–5). The pig heart exhibits a markedly restricted innate collateral circulation, analogous to that observed in the human heart (6).

Myocardial infarction (MI) can be induced in porcine models by partial occlusion of the coronary artery, either by open or closed chest procedures. The most appropriate method for modeling the most commonly occurring MI in humans is to use a closed-chest, balloon ischemia/reperfusion model. MI results in myocardial cell death and perfusion imbalance. Biochemical markers can demonstrate myocardial ischemia. In 1954, serum glutamic oxaloacetic acid transaminase (SGOT), now known as aspartate aminotransferase (AST), was the first biochemical marker for the diagnosis of AMI (7, 8), and its elevation correlates with the size of the heart infarct (9). Acute circulatory changes, induced by AMI, directly affect hepatic perfusion and metabolic activity (10–12).

Perfusion dysfunction leads to hepatocyte necrosis, which, as the primary source of ALT, causes its elevation. Despite the fact that the usefulness of aminotransferases in the diagnosis of AMI is decreasing due to the emergence of more specific biomarkers of myocardial damage, such as hs-cTn, they remain valuable diagnostic parameters. This is corroborated by the findings of recently published studies which demonstrate a correlation between elevated aminotransferase activity in the acute phase of AMI and subsequent prognosis. The AST/ALT ratio (hereafter referred to as De Ritis ratio) has prognostic value in patients with acute myocardial infarction (13).

The objective of our research is to evaluate the alterations in biochemical parameters throughout the adaptation of the Hungarian Pannon minipig myocardial infarction model and construct a database of standard clinical baseline values, blood parameters, biometric data, and cardiac function obtained from cardiovascular studies in experimental animals, which will be incorporated into the breeding database.

## 2 Materials and methods

The protocol and ethical guidelines for this experiment were approved and controlled by the Government Office for Somogy County under the license number SOI/31/00161-7/2020.

### 2.1 Animals

Twenty-four intact mature female Pannon minipig (Source of supply: Farm registered in the Hungarian state Farm Animal Identification System (ENAR)) from authority-monitored breeders were included in the study. The mean body weights were 60 kg at the mean age of 9 months at order. The animals were free from Aujeszky disease, PRRS, Leptospirosis, and Brucellosis, approved by the EU and Hungarian authority monitoring system. The animals were placed in groups of 6–7 animals. By keeping them in a traditional pen, we provided the possibility of expressing natural behaviors, preventing social stress caused by isolation. Abundant space and environmental enrichment were also provided to allow for more physical activity in the daily activity pattern of these animals, compared to those kept in individual cages. Throughout the experiment, the animals were fed a special diet containing 88.2% dry matter, 14.01% crude protein, 2.57% crude fat, 10.13% crude fiber, and 11.35 MJ/kg digestible energy (“Agroszász” Experimental Pig Food; Szászvár, Hungary). The minipigs were fed an amount equal to 1.5% of their body weight twice per day, with drinking water available *ad libitum*.

Animals were observed and checked for signs of illness, distress, or deviation from normal (activity, food intake (qualitative), etc.). No abnormalities and/or unexpected observations were reported in individual animal records. The animals were observed individually before and after the procedure (on day 0). Daily farm-side observations were made to check the animals, with a focus on behavioral changes or illness. The animals were placed in groups of 6–7 animals. During the 30-day follow-up period, the animals were under regular veterinary control, and no interventions were needed. The animals were transported to the Institute in small groups 1 day before the study started. In the research area, they were randomly placed in individual cages and signed with a simply growing number sequence. The animals were kept individually until the third day of the experiment, to perform the individual medications and treatments. The isolation time was 4 days. Blinding was not considered during the study. The animals underwent the same procedure without treatment, and the experimental unit was the single animal. At the endpoint of the experiment on day 30, the animals were gently anesthetized by inhalation of a 5% volume isoflurane and were euthanized by administration of 30 mg/kg of body weight potassium chloride into the jugular vein and then dissected.

### 2.2 Cardiac magnetic resonance imaging (cMRI) examinations

Cardiac MRI examinations were performed 24–48 h before the induction of the myocardial infarction. The animals were taken to the institute 1 day before the MRI, which was performed after 12 h of fasting. Before the MRI scan, the minipigs were sedated with a combination of 12 mg/kg ketamine hydrochloride (Narkamon 100 mg/mL inj., Bioveta; Ivanovice na Hané, Czechia), 1 mg/kg xylazine (CP-Xylazin2% inj., CP-Pharma GmbH; Burgdorf, Germany), and 0.04 mg/kg atropine sulfate (Atropine sulfuricum-EGIS 1 mg/mL inj., Egis; Budapest, Hungary) administered intramuscularly, followed by the placement of an endotracheal tube (Chanelmed Ltd.; diameter: 5.0–6.0). Imaging was performed under inhalation anesthesia with 1.5–2.5 m/m% isoflurane (Isoflutek 1,000 mg/g, Karizoo; Barcelona, Spain) and 2 L/min oxygen with a MAGNETOM Vision Plus 1.5 T MRI scanner (Siemens AG; Erlangen, Germany) with ECG control. To improve image quality, cine MRI images in short-axis and long-axis views of the heart were acquired following the intravenous administration of 1.5 mg/kg atracurium (Tracrium 10 mg/mL inj., GlaxoSmithKline; Brentford, United Kingdom) and mechanical ventilation (10 mL/kg 15/min). The animals were kept in single cages until the control MRI scans on day 3. After the MRI scan on day 3, the animals were transported to their usual keeping place and kept in groups. The control MRI scans were performed on day 30, and the animals were transported back to the Institute 1 day before the procedure. Preparation and method of examination were the same for the MRI imaging performed on days 3 and 30. At these check points, gadobutrol (Gadovist 1 mmoL/mL inj., Bayer; Leverkusen, Germany) was administered intravenously at a dose of 0.16 mmol/kg for the examination of the myocardium, the determination of left ventricular function, and the measurement of perfusion and viability.

### 2.3 Infarction induction

The day before AMI induction, the animals were treated with 600 mg clopidogrel and 300 mg aspirin per os in the drinking water, followed by a daily dose of 100 mg acetylsalicylic acid (Aspirin protect 100 mg tablets, Bayer; Leverkusen, Germany) and 75 mg clopidogrel (Trombex 75 mg tablets, GlaxoSmithKline; Brentford, United Kingdom) from day 1, administered per os. Induction of AMI was performed on day 0 under general anesthesia as described for the acquisition of MRI images. Surgical access was provided to the femoral artery and the jugular vein, and a sheath (St. Jude Medical introducer set) was inserted (6–7 F diameter, according to the size). After blood was drawn, 5,000 IU heparin was administered via the sheath following blood sampling. AMI induction was performed under invasive hemodynamic monitoring and by placing a balloon catheter (2.5–2.75 mm diameter, 8–15 mm length) in the left anterior descending coronary artery (LAD) after the origin of the second diagonal branch and inflating the balloon with 5–6 atm for 90 min, followed by reperfusion. A Siemens Cios Alpha C-arm, a Siemens Axiom Sensis hemodynamic unit, and a Comen C-50 V patient monitor were used during the angiographic procedure. The contrast material used was Xenetix 350 mg I/ml (iobitridol); Guerbet, and contrast volumes range from 50 to 100 mL/pig (245–370 mg/kg with a short injection duration).

Reperfusion and ventricular function were confirmed by angiography, and a second blood sample was taken from the sheath. The sheath was then removed, the femoral artery was ligated, and the wound was closed. The surgical site was treated with an aluminum-containing polymer spray and a broad-spectrum antibiotic (procain-benzylpenicillin and benzathine-benzylpenicillin 5-5-mg/kg and dihydrostreptomycin 10 mg/kg) (Shotapen inj., Virbac; Carros, France), and a long-acting, non-steroidal anti-inflammatory and pain killer drug (0.4 mg/kg meloxicam) (Meloxidyl inj., Ceva; Marseille, France) was administered. The animals were allowed to recover under continuous monitoring and were extubated when the swallowing reflex returned. After extubation, the animals were placed in individual cages in a climate-controlled (23°C) surgical preparation room until full recovery.

### 2.4 Blood sampling

**Figure 1:**
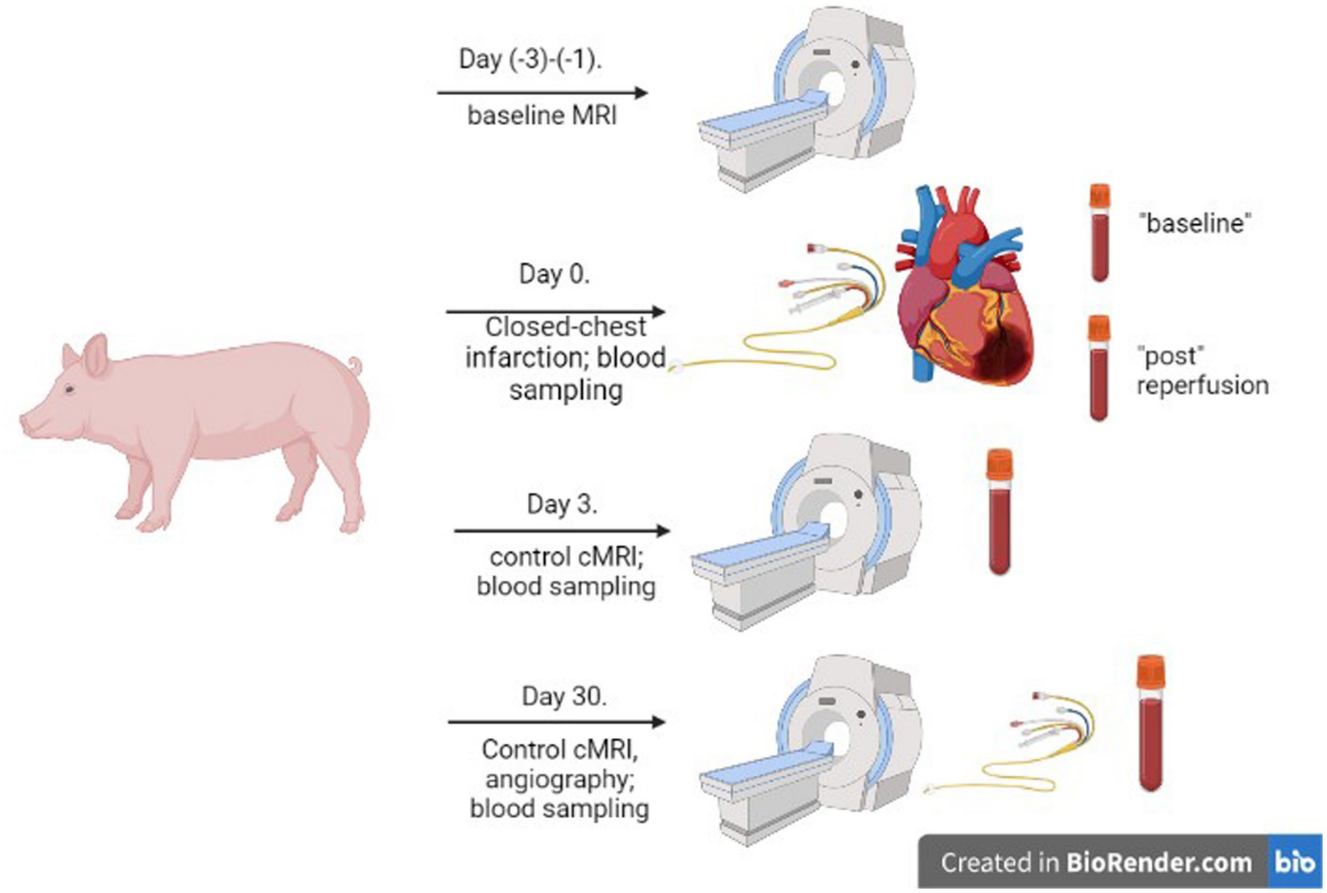
Experimental design. A schematic diagram summarizes the experimental procedures in the minipig myocardial infarction model.

Blood samples were obtained from the animals from the jugular vein at different stages of the myocardial infarction (0: before infarction-induction; 1: post-reperfusion; 2: 72 h; 3: day 30). The experimental design is shown in Figure 1. Under anesthesia, 20 mL of blood was drawn aseptically from the sheath in the jugular vein and equally distributed into blood collection tubes, one of them containing ethylenediaminetetraacetic acid (EDTA) and the other without any additives (serum tube). The baseline samples were collected immediately after the introducer implantation, ca. 15–30 min before the AMI induction. The follow-up samples were taken after the MR imaging procedure.

The control MRI was followed by surgical preparation as described for the AMI induction procedure, and 20 mL of blood samples were obtained again from the animals through a sheath. Blood samples were centrifuged for 10 min at 4,000 rpm, and the plasma or serum was transferred to storage tubes and stored at −20°C in quality-controlled refrigerators. The blood samples were tested at the veterinary laboratory Praxis Lab Ltd., and the following biochemical parameters were determined: AST, ALT, LDH, CK, AST/ALT ratio, and high-sensitivity troponin I. Indicators of sample quality (hemolysis, icterus, and lipemia) were evaluated. Measurement of AST, ALT, LDH, and CK was carried out on a Beckman Coulter AU480 autoanalyzer using dedicated reagent kits (Beckman Coulter, Brae, CA, United States). Troponin I was determined using a high-sensitive human electro-chemiluminescent immunoassay (Acces hsTnI; B52699) on a Beckman Coulter Access 2 analyzer. The used materials and reagents were validated for swine species.

Basic hematological and biochemical data were collected prior to the study, analyzing blood samples collected from 35 healthy pigs of the herd. The results were identical to the meat-type Landrace pig’s laboratory normal values.

### 2.5 Statistical analysis

The animals were included in the study with a successful LAD occlusion-reperfusion process and evaluable MR images at all three time points. Animals were excluded when were not suitable for the procedure or died before or during the catheterization.

To observe differences between the time points, linear mixed effect models were used. The individual ID of the animals was used as a random factor, and no other factors were used for adjusting. For comparison, marginal means were estimated using the restricted maximum likelihood (REML) fitting method. For *p*-value calculation, Satterthwaite’s method was applied. Due to the non-normal nature of the examined blood parameters, the data were logarithmized to perform the calculations. When the results are reported, the data are transformed back and reported as mean (M) and standard deviation (SD). Bonferroni–Holm *post-hoc* test was used to compare different time points to control the family-wise error rate (FWER). A result was considered significant if *p* < 0.05. All calculations were made using R statistical software^1^ (14) and packages lme4 (15) and lmerTest (16).

## 3 Results

Six animals died during the experiment, which were excluded from the study, and 18 pigs were followed until day 30.

### 3.1 AST

**Figure 2:**
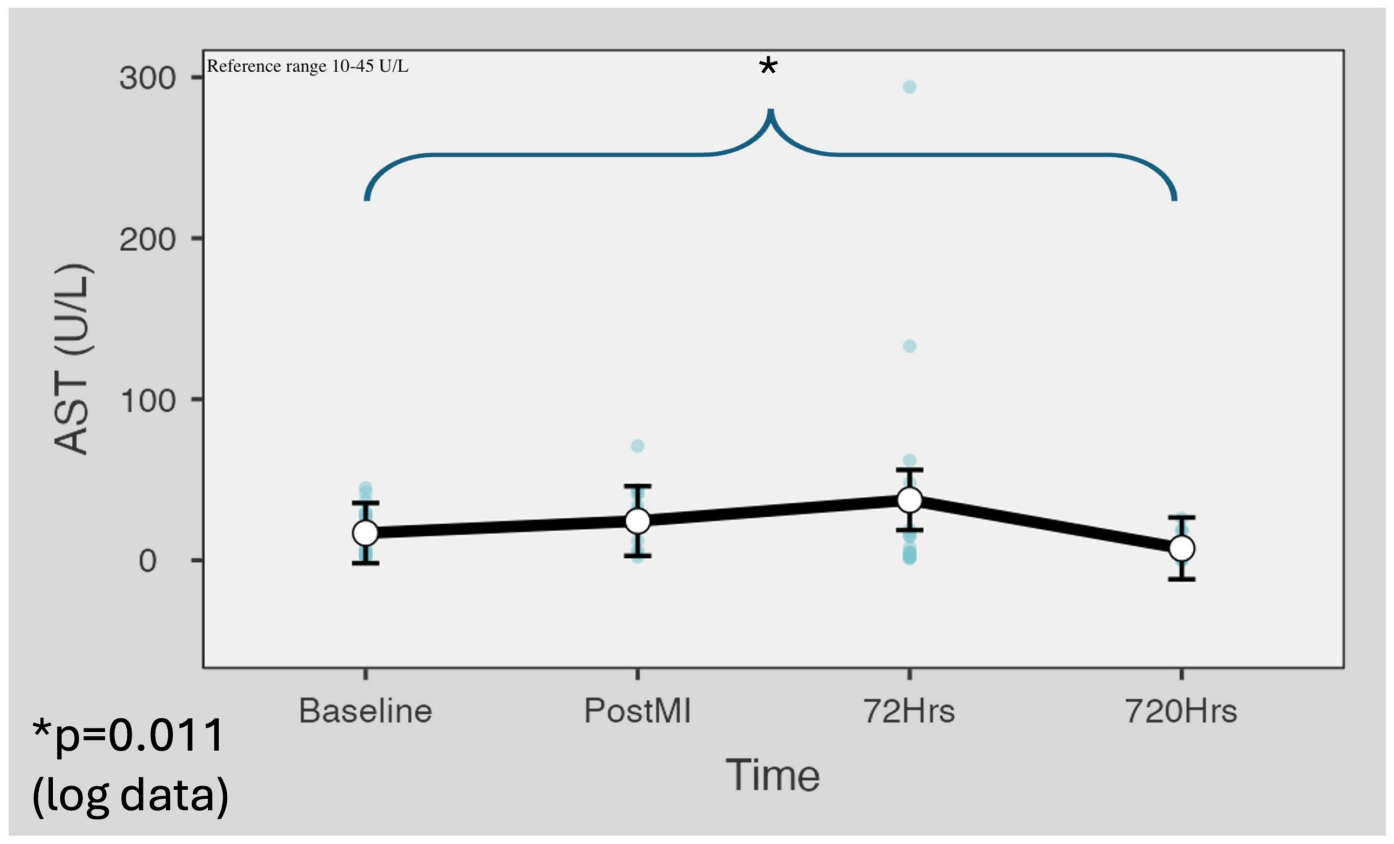
AST level change in a minipig myocardial infarction model. The mean AST levels were within the reference range (10–45 U/L) at baseline (M = 16.89 U/L, SD = 15.61). The post-reperfusion (M = 27.38 U/L, SD = 19.56) and 72 h (M = 37.4 U/L, SD = 71.87) blood results did not differ statistically (*p* = 0.532, *p* = 0.884, respectively). The day 30 values were lower than the baseline (M = 8 U/L, SD = 7.66). Marginal means were estimated with 95% confidence intervals using the restricted maximum likelihood fitting method. For *p*-value calculation, Satterthwaite’s method was applied to the results of logarithmic data. AST, aspartate transaminase.

The mean AST levels were within the reference range (10–45 U/L) at baseline (M = 16.89 U/L, SD = 15.61). The post-reperfusion (M = 27.38 U/L, SD = 19.56) and 72 h (M = 37.4 U/L, SD = 71.87) blood results did not differ statistically (*p* = 0.532, *p* = 0.884, respectively). The day 30 values were lower than the baseline (M = 8 U/L, SD = 7.66), and the difference was statistically significant (*p* = 0.011) (Figure 2).

### 3.2 ALT

**Figure 3:**
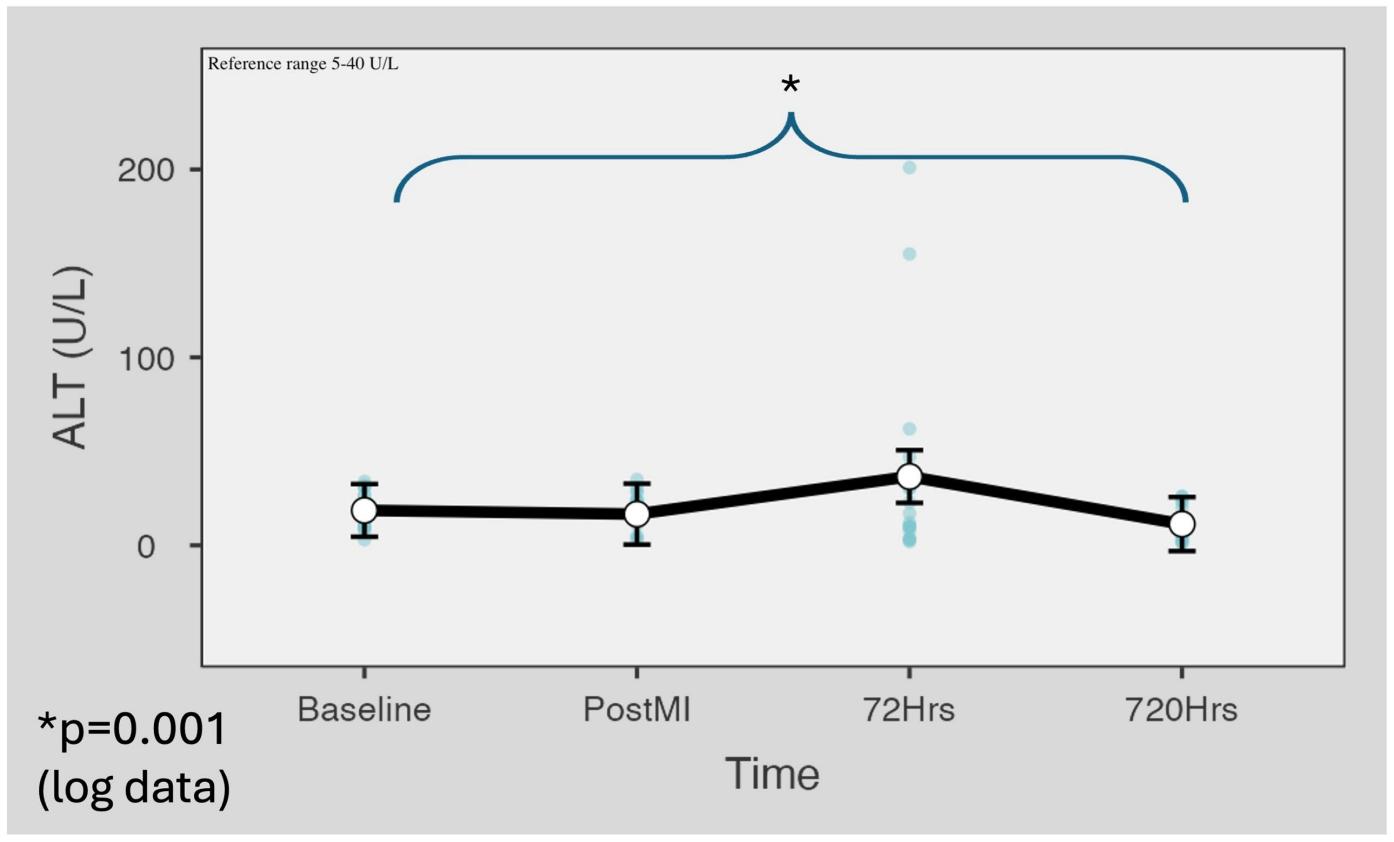
Kinetic of the ALT values in the minipig infarction model. ALT levels were within the physiological reference range values in the baseline samples taken before AMI induction (M = 18.6 U/L SD = 9.54) and immediately following reperfusion (M = 19.3 U/L, SD = 10.99). The mean value increased not significantly (M = 36.6 U/L, SD = 54.52, *p* = 0.873) in samples obtained at 72 h. The mean of the results obtained from blood samples collected on day 30 was statistically lower (*p* = 0.001) than the baseline values (M = 11.7 U/L, SD = 9.08). Marginal means were estimated with 95% confidence intervals using the restricted maximum likelihood fitting method. For *p*-value calculation, Satterthwaite’s method was applied to the results of logarithmic data. ALT, alanine aminotransferase.

**Figure 4:**
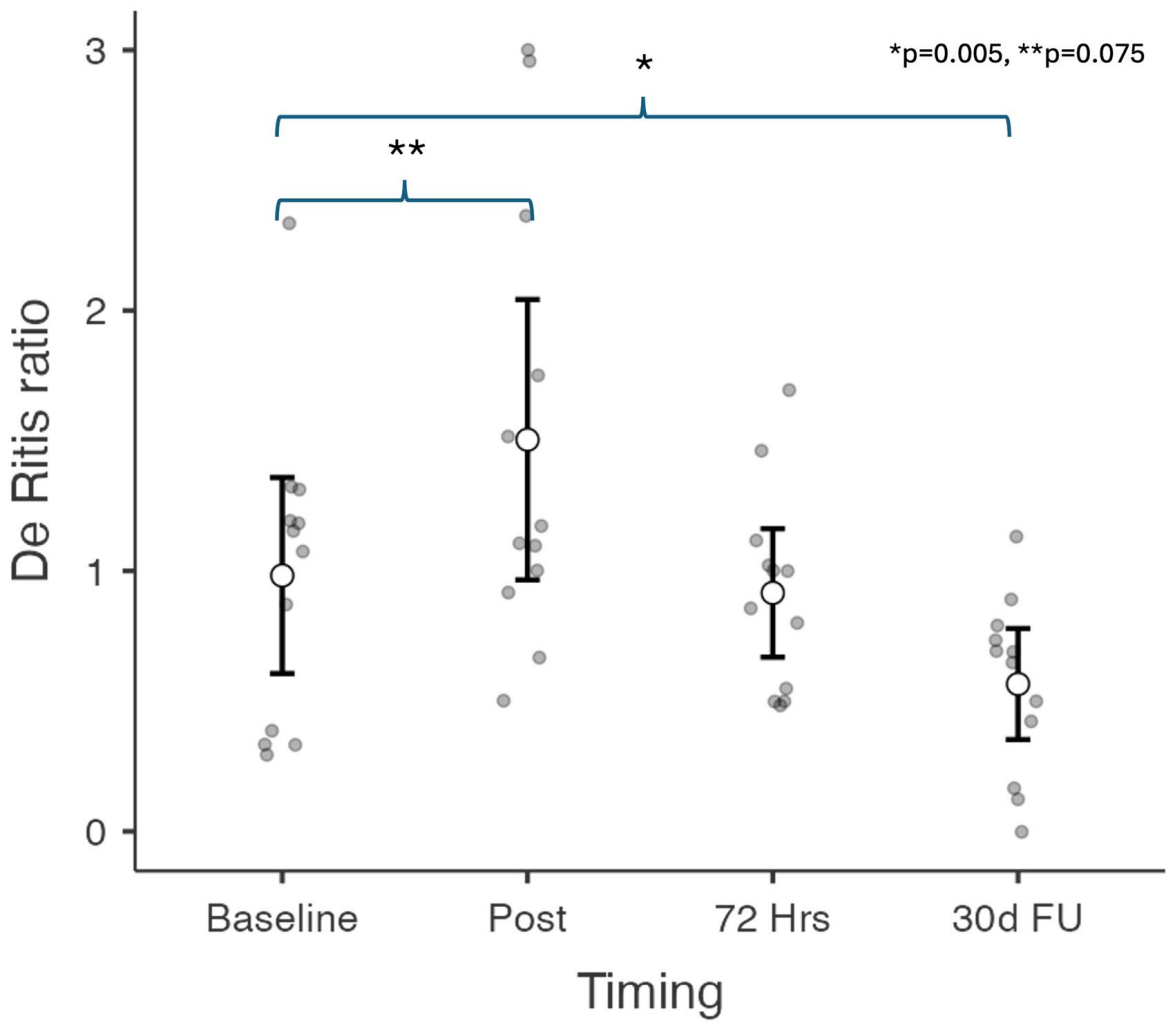
AST/ALT (De Ritis) ratio values in the 30-day minipig infarction model. In the AST/ALT ratio was a statistically significant (*p* = 0.005) difference between the baseline and day 30 values. Data are presented as estimated marginal means. Significance is calculated from the results of logarithmic data.

ALT levels were within the physiological reference range values (5–40 U/L) in the baseline samples taken before AMI induction (M = 18.6 U/L SD = 9.54) and in the samples taken immediately following reperfusion (M = 19.3 U/L, SD = 10.99). The mean value increased not significantly with great individual variation (M = 36.6 U/L, SD = 54.52, *p* = 0.873) for the samples obtained at 72 h. The mean of the results obtained from blood samples collected on day 30 was statistically lower (*p* = 0.001) than the baseline values (M = 11.7 U/L, SD = 9.08) (Figure 3). In the AST/ALT ratio was a statistically significant (*p* = 0.005) difference between the baseline and day 30 values (Figure 4).

### 3.3 LDH

**Figure 5:**
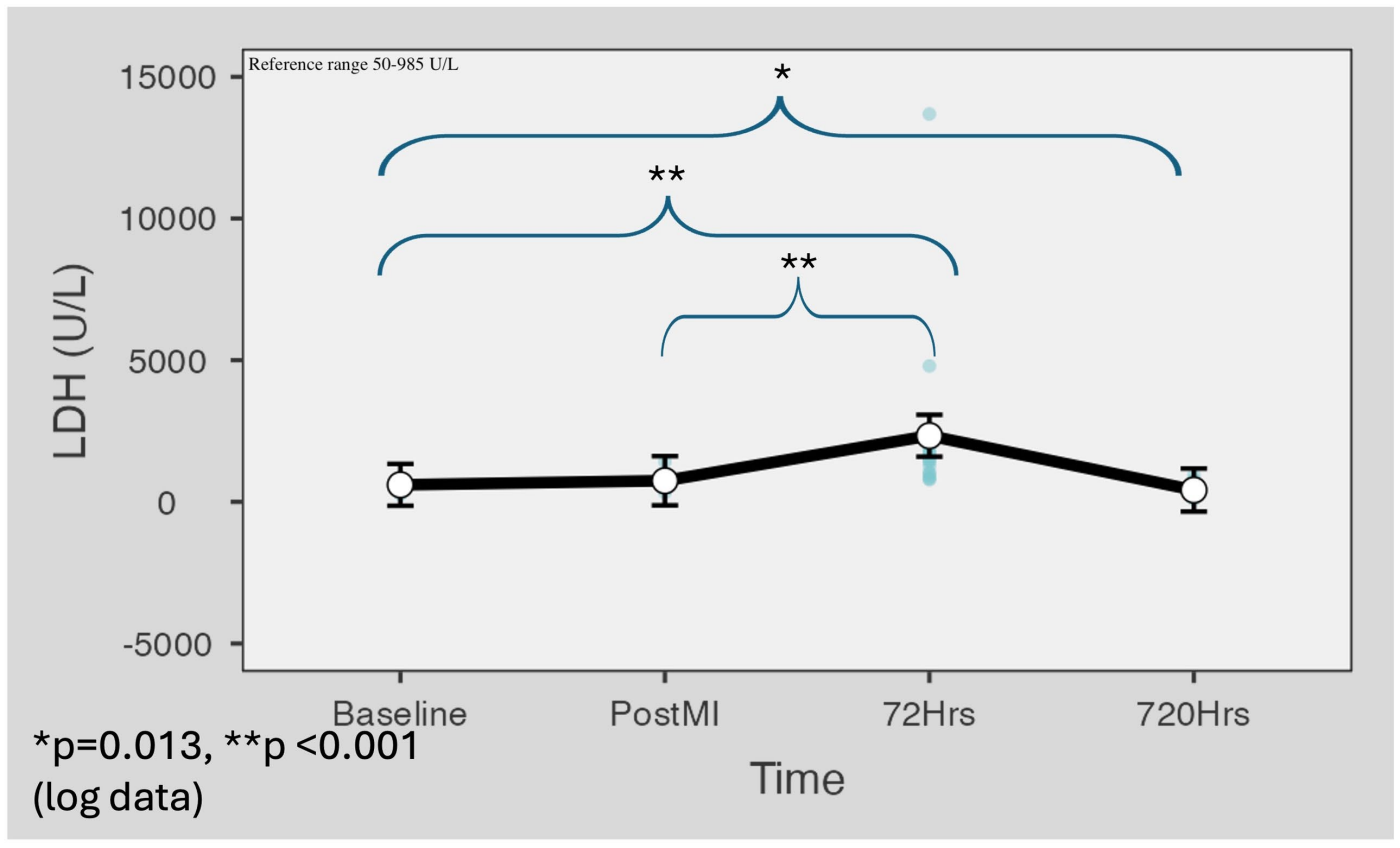
LDH level changes in the minipig myocardial infarction model. LDH mean serum levels were increased in the 72-h samples (M = 2,334 U/L, SD = 2,970) *p* < 0.001 compared to baseline (M = 601 U/L, SD = 208) and post-reperfusion (M = 768 U/L, SD = 264) and were above the reference range values. Values dropped to a level significantly below the baseline values on day 30 (M = 420 U/L, SD = 210) (*p* = 0.013). Marginal means were estimated with 95% confidence intervals using the restricted maximum likelihood fitting method. For *p*-value calculation, Satterthwaite’s method was applied to the results of logarithmic data. LDH, lactate dehydrogenase.

LDH mean serum levels were markedly increased in the 72-h samples (M = 2,334 U/L, SD = 2,970) (*p* < 0.001) compared to baseline (M = 601 U/L, SD = 208) and post-reperfusion (M = 768 U/L, SD = 264) and were above the reference range values (50–985 U/L) for porcine. LDH values dropped to a level significantly below the baseline values on day 30 (M = 420 U/L, SD = 210) (*p* = 0.013) (Figure 5).

### 3.4 CK

**Figure 6:**
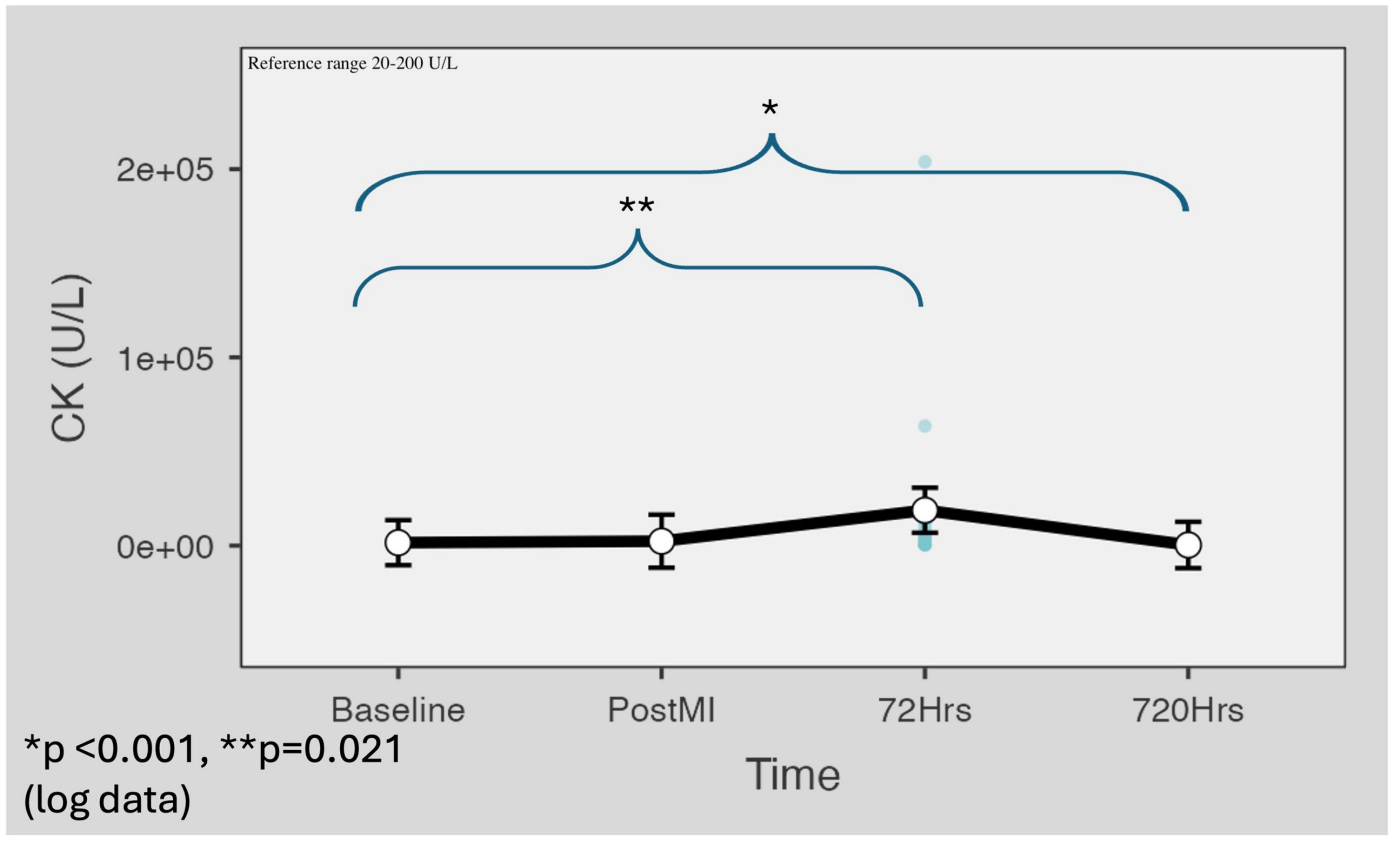
CK value changes in the minipig infarction model. The CK levels were above the reference range at the first blood sampling time point (M = 1,606 U/L, SD = 832). An increasing trend was observed for the post-reperfusion (M = 2,400 U/L, SD = 844, *p* = 0.389) and the 72-h (M = 2,349 U/L, SD = 2,300, *p* = 0.021) samples. The mean of the day 30 values (M = 374 U/L, SD = 201) was lower (*p* < 0.001) than the baseline values. Marginal means were estimated with 95% confidence intervals using the restricted maximum likelihood fitting method. For *p*-value calculation, Satterthwaite’s method was applied to the results of logarithmic data. CK, creatine kinase.

The CK levels were above the reference range values already at the first blood sampling time point (M = 1,606 U/L, SD = 832). An increasing trend was observed for the post-reperfusion (M = 2,400 U/L, SD = 844, *p* = 0.389) and the 72-h (M = 2,349 U/L, SD = 2,300, *p* = 0.021) samples. The mean of the day 30 values (M = 374 U/L, SD = 201) was lower (*p* < 0.001) than the baseline values. The CK reference range for swine is 20–200 U/L (Figure 6).

### 3.5 Troponin I

**Figure 7:**
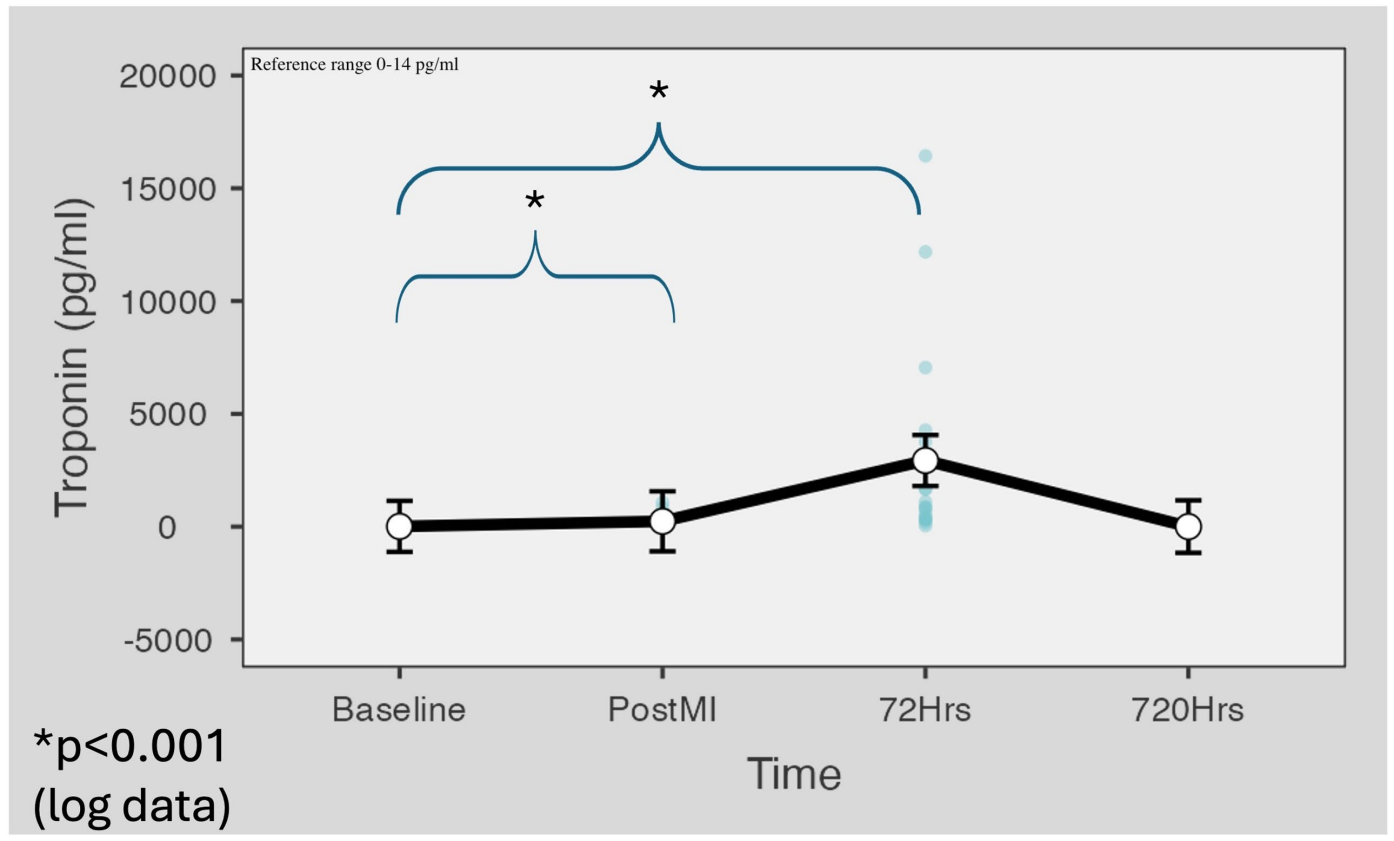
Cardiac troponin value changes during the minipig infarction model. The baseline troponin I mean value was 12.1 pg./mL. Troponin I mean values were significantly increased immediately following AMI (M = 237.2 pg./mL, SD = 354.7, *p* < 0.001) and increased manifold by the 72 h (M = 2,933 pg./mL, SD = 4,573, p < 0.001). By day 30, the troponin levels dropped to a normal level (M = 19.8 pg./mL, SD = 70.4, *p* = 0.426). Marginal means were estimated with 95% confidence intervals using the restricted maximum likelihood fitting method. For *p*-value calculation, Satterthwaite’s method was applied to the results of logarithmic data.

The baseline troponin I mean value was 12.1 pg./mL. Troponin I mean values were significantly increased immediately following AMI (M = 237.2 pg./mL, SD = 354.7, p < 0.001) and increased manifold by the 72 h (M = 2,933 pg./mL, SD = 4,573, *p* < 0.001). By day 30, the troponin levels dropped to a normal level, to almost zero (M = 19.8 pg./mL, SD = 70.4, *p* = 0.426), and did not differ from the results of the baseline level (Figure 7). The detailed statistical analysis is demonstrated in the Supplementary material.

## 4 Discussion

The objective of the present study was to assess the alterations in biochemical parameters throughout the adaptation of the Pannon minipig model in myocardial infarction. It is suggested that the peri-procedural environment and stress factors, in addition to the induction of myocardial infarction, also influenced the kinetics of cardiac biomarkers. The methodology used raises the possibility of inappropriate acclimatization processes, which significantly impacted some biochemical parameters. The stress-associated changes in the immune-, endocrine-, cardiovascular, and central nervous systems can affect the homeostasis of experimental animals. In preclinical studies, aiming to model human pathological changes, it is crucial to the welfare and stability of the experimental unit to achieve reliable and reproducible results. With this in mind, particular care should be taken to minimize the negative effects affecting the animals and standardize the experimental interventions.

The animals were maintained at the experimental site for approximately 72 h before baseline blood samples were collected. The animals were housed in individual cages, administered medication, and underwent a series of MRI scans. In contrast, on day 30, the animals were at the experimental site only for 24 h, and blood samples were taken immediately following the single MRI scan, during the same anesthetic procedure. The animals underwent similar medical procedures, with the only difference of time spent in individual cages. The increased baseline values can be associated with physical exposure (injections, minor injuries to skeletal muscles, and physical strain) and significant social and other stressors. This emphasizes the importance of considering the stress sensitivity of pigs when designing and interpreting animal model studies. It is crucial to determine the optimal times for blood collection and prioritize animal welfare.

The baseline and 30-day values are both within or below the reference range, even if there is a statistically significant difference between the group means at these time points. This could be due to random biological variation and the analytical method variance. The same trend was observed for multiple markers, supporting the differences between the measurement time points.

The less specific cardiac biomarkers (ALT, AST) and the AST/ALT ratio showed significant differences between the baseline and day 30 timepoints, which can affect the prognosis. In our study, these levels were likely influenced by several factors including intramuscular drug administration, minor surgery for sheet insertion, and drug-induced liver injury. The kinetics of cardiac troponin, CK, and LDH reflected the known literature in relation to myocardial necrosis (17). On day 30, the results were significantly lower than baseline values for the majority of biomarkers.

By day 30, troponin I level had returned to baseline values near zero, which can be attributed to its rapid kinetics (18, 19). In contrast, the higher baseline levels of biomarkers with slower kinetics compared to 30-day results may be due to the experimental methods, which have the potential to raise animal welfare concerns (20).

Cardiac-specific troponin I (cTnI) is considered the “gold-standard” diagnostic biomarker of acute myocardial injury as it is exclusively found in the myocardium and released from necrotic myocardial tissue. For the first time, high-sensitivity cTn assays enabled the accurate quantification of cardiomyocyte injury around the 99th percentile, thereby significantly increasing the accuracy of MI detection (21). Blood troponin levels are proportional to the extent of tissue damage. In swine—just as in humans—serum troponin levels can be used to indicate ischemic damage of the myocardium (19, 22, 23). The specificity of hs-troponin has been confirmed in minipigs, thereby establishing it as the superior choice over the CK-MB isoenzyme (20). In our study, a significant increase in troponin levels beyond 24 h was attributed to acute heart failure, which may cause an increase in short- and long-term mortality (24). This is supported by the fact that left ventricular function measured by invasive method showed a significant decrease immediately after infarction, but despite a troponin level of 10 times at 72 h, both cardiac MRI and invasive methodology demonstrated a more compensated status after the acute phase (25).

The study is subject to certain limitations. These include the necessity to adhere to the 3Rs as a guiding concept, and the fact that the National Scientific Ethics Committee for Animal Experiments places severe restrictions on the number of animals that can be used in experiments. Due to the limited number of animals available, only female animals were included in the study to minimize the potential confounding effect of sex hormones on the results. It was not possible to create a sham group; therefore, each animal served as its control. Although CK-MB levels were not determined, troponin I measurements were used to confirm the effect of infarct induction.

## Data Availability

The original contributions presented in the study are included in the article/Supplementary material, further inquiries can be directed to the corresponding author.

## Footnotes

^1^ https://www.R-project.org/
