# Supplementary material for: Blood biochemistry changes in a minipig infarction model
Source: Front Vet Sci. 2025 Jan 27;12:1493660. doi: 10.3389/fvets.2025.1493660 (PMC11807996; doi:10.3389/fvets.2025.1493660)
Supplement: Supplementary file 1 [file Data_Sheet_1.pdf]

Supplementum

1 Mixed Model – AST level

Model Info

Info	
Estimate	Linear mixed model fit by REML
Call	AST - Transform 5 ~ 1 + Time+(1 | ID)
AIC	76.888
BIC	101.079
LogLikel.	-38.016
R-squared Marginal	0.092
R-squared Conditional	0.743
Converged	yes
Optimizer	bobyqa

1.1 Model Results

Fixed Effect Omnibus tests

	F	Num df	Den df	p
Time	7.303	3	44.361	0.00044

Note. Satterthwaite method for degrees of freedom

Fixed Effects Parameter Estimates

Names	Effect	Estimate	SE	95% Confidence Interval		df	t	p
				Lower	Upper			
(Intercept)	(Intercept)	0.951	0.118	0.733	1.169	17.054	8.029	< .00001
Time1	PostMI - Baseline	0.153	0.111	-0.061	0.354	44.539	1.372	0.17706
Time2	72Hrs - Baseline	0.066	0.100	-0.132	0.268	44.002	0.665	0.50953
Time3	720Hrs - Baseline	-0.330	0.104	-0.538	-0.140	44.204	-3.187	0.00264

Random Components

Groups	Name	SD	Variance	ICC
ID	(Intercept)	0.476	0.226	0.717
Residual		0.299	0.089	

Note. Number of Obs: 65 , groups: ID 18

1.2 Post Hoc Tests

Post Hoc Comparisons - Time

Comparison							
Time		Time	Difference	SE	t	df	p_{holm}
72Hrs	-	720Hrs	0.396	0.104	3.825	44.205	0.00204
Baseline	-	720Hrs	0.330	0.104	3.185	44.205	0.01060

Post Hoc Comparisons - Time

Comparison							
Time		Time	Difference	SE	t	df	p_{holm}
Baseline	-	72Hrs	-0.066	0.100	-0.665	44.004	0.88364
Baseline	-	PostMI	-0.153	0.111	-1.370	44.541	0.53242
PostMI	-	720Hrs	0.483	0.116	4.166	44.855	0.00084
PostMI	-	72Hrs	0.087	0.111	0.776	44.541	0.88364

1.3 Estimated Marginal Means

Time

				95% Confidence Interval	
Time	Mean	SE	df	Lower	Upper
Baseline	0.978	0.132	26.081	0.706	1.251
PostMI	1.131	0.142	32.216	0.843	1.419
72Hrs	1.045	0.132	26.081	0.772	1.317
720Hrs	0.648	0.135	28.102	0.371	0.926

1.4 Effects Plots

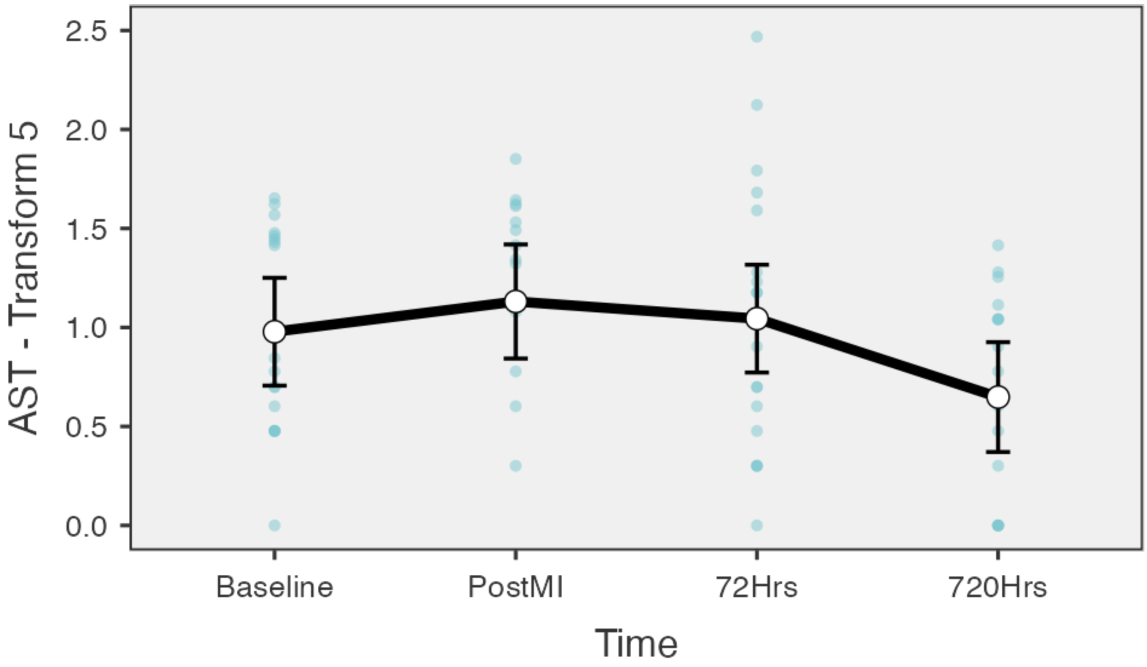

1.5 Assumption Checks

1.5.1 Q-Q Plot

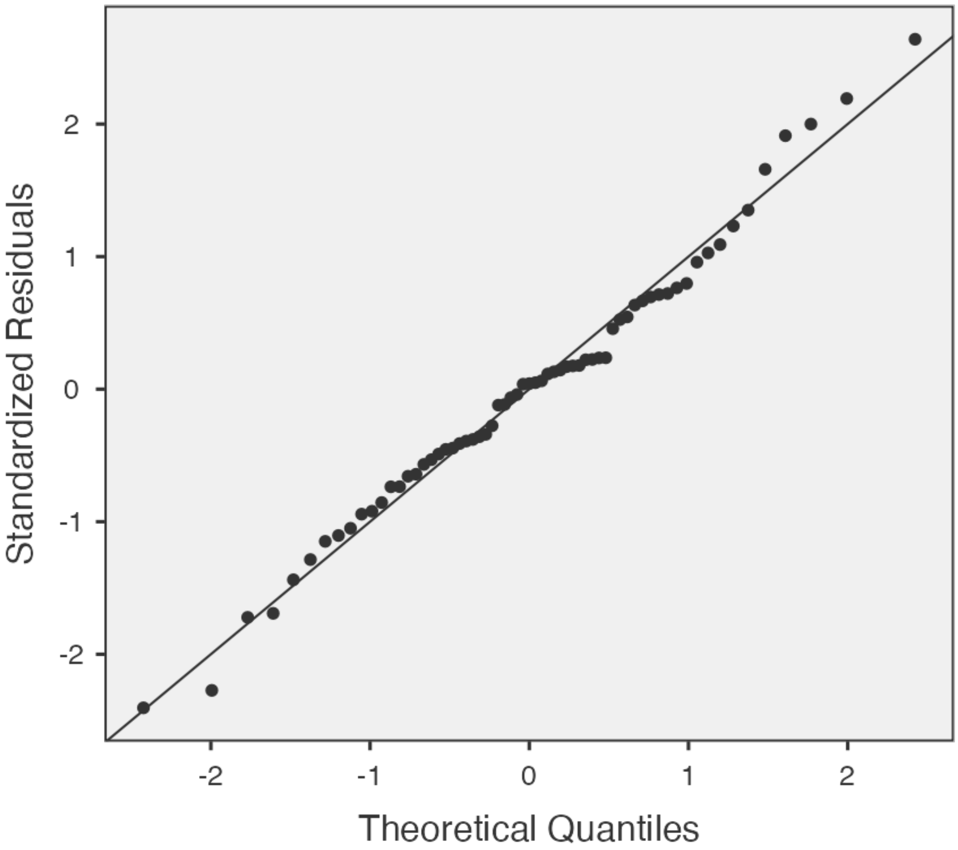

2 Mixed Model – ALT level

Model Info

Info	
Estimate	Linear mixed model fit by REML
Call	ALT - Transform 4 ~ 1 + Time+(1 | ID)
AIC	42.001
BIC	68.498
LogLikel.	-21.680
R-squared Marginal	0.090
R-squared Conditional	0.740
Converged	yes
Optimizer	bobyqa

2.1 Model Results

Fixed Effect Omnibus tests

	F	Num df	Den df	p
Time	7.448	3	45.362	0.00037

Note. Satterthwaite method for degrees of freedom

Fixed Effects Parameter Estimates

				95% Confidence Interval				
Names	Effect	Estimate	SE	Lower	Upper	df	t	p
(Intercept)	(Intercept)	1.095	0.090	0.892	1.276	17.119	12.184	< .00001
Time1	PostMI - Baseline	-0.128	0.085	-0.299	0.059	45.601	-1.504	0.13938
Time2	72Hrs - Baseline	0.012	0.076	-0.137	0.162	45.057	0.160	0.87335
Time3	720Hrs - Baseline	-0.312	0.078	-0.468	-0.163	45.157	-4.016	0.00022

Random Components

Groups	Name	SD	Variance	ICC
ID	(Intercept)	0.361	0.130	0.714
Residual		0.229	0.052	

Note. Number of Obs: 66 , groups: ID 18

2.2 Post Hoc Tests

Post Hoc Comparisons - Time

Comparison							
Time		Time	Difference	SE	t	df	p_{holm}
72Hrs	-	720Hrs	0.324	0.078	4.173	45.104	0.00081
Baseline	-	720Hrs	0.312	0.078	4.016	45.104	0.00111

Post Hoc Comparisons - Time

Comparison							
Time		Time	Difference	SE	t	df	p_{holm}
Baseline	-	72Hrs	-0.012	0.076	-0.160	45.004	0.87335
Baseline	-	PostMI	0.128	0.085	1.503	45.548	0.31980
PostMI	-	720Hrs	0.184	0.087	2.115	45.706	0.15969
PostMI	-	72Hrs	-0.140	0.085	-1.646	45.548	0.31980

2.3 Estimated Marginal Means

Time

				95% Confidence Interval	
Time	Mean	SE	df	Lower	Upper
Baseline	1.202	0.101	26.392	0.995	1.409
PostMI	1.073	0.108	32.643	0.854	1.293
72Hrs	1.214	0.101	26.392	1.007	1.421
720Hrs	0.890	0.102	27.363	0.681	1.099

2.4 Effects Plots

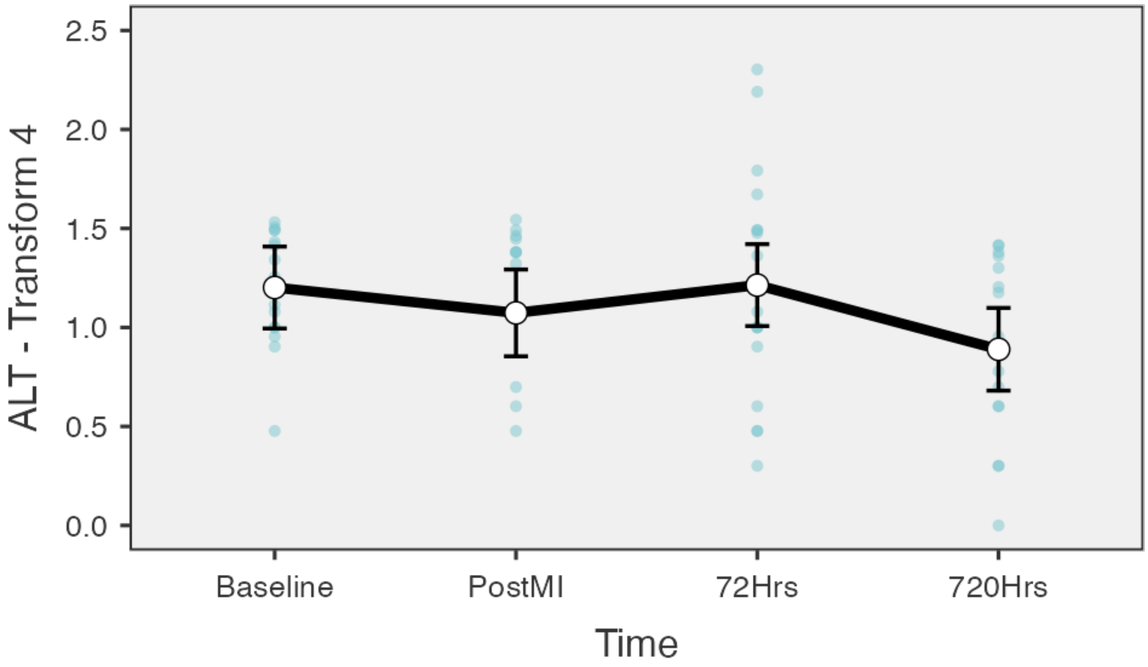

2.5 Assumption Checks

2.5.1 Q-Q Plot

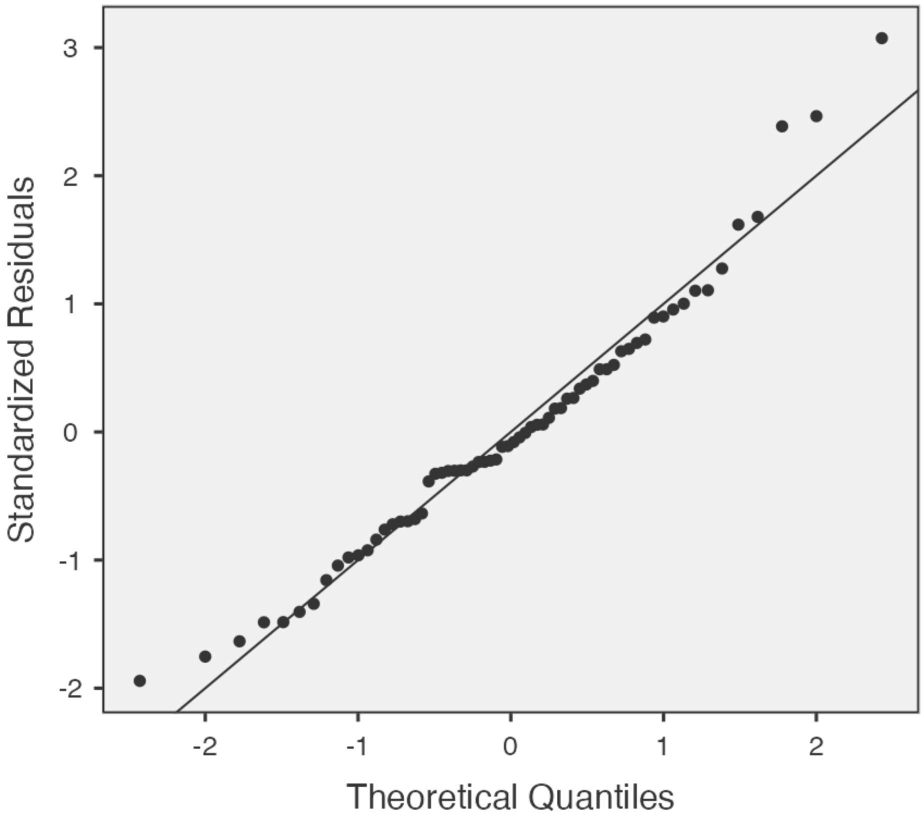

3 Mixed Model – LDH level

Model Info

Info	
Estimate	Linear mixed model fit by REML
Call	LDH - Transform 1 ~ 1 + Time+(1 | ID)
AIC	-7.419
BIC	21.979
LogLikel.	1.579
R-squared Marginal	0.554
R-squared Conditional	0.690
Converged	yes
Optimizer	bobyqa

3.1 Model Results

Fixed Effect Omnibus tests

	F	Num df	Den df	p
Time	38.622	3	45.702	< .00001

Note. Satterthwaite method for degrees of freedom

Fixed Effects Parameter Estimates

Names	Effect	Estimate	SE	95% Confidence Interval Lower	95% Confidence Interval Upper	df	t	p
(Intercept)	(Intercept)	2.843	0.038	2.777	2.916	16.734	75.098	< .00001
Time1	PostMI - Baseline	0.077	0.070	-0.070	0.228	46.576	1.104	0.27508
Time2	72Hrs - Baseline	0.480	0.063	0.351	0.600	44.645	7.615	< .00001
Time3	720Hrs - Baseline	-0.184	0.064	-0.319	-0.069	44.994	-2.869	0.00625

Random Components

Groups	Name	SD	Variance	ICC
ID	(Intercept)	0.125	0.016	0.305
Residual		0.189	0.036	

Note. Number of Obs: 66 , groups: ID 18

3.2 Post Hoc Tests

Post Hoc Comparisons - Time

Comparison Time		Comparison Time	Difference	SE	t	df	p_{holm}
72Hrs	-	720Hrs	0.664	0.064	10.348	45.394	< .00001
Baseline	-	720Hrs	0.184	0.064	2.867	45.394	0.01252

Post Hoc Comparisons - Time

Comparison							
Time		Time	Difference	SE	t	df	p_{holm}
Baseline	-	72Hrs	-0.480	0.063	-7.615	45.050	< .00001
Baseline	-	PostMI	-0.077	0.070	-1.101	46.951	0.27652
PostMI	-	720Hrs	0.261	0.071	3.663	47.468	0.00188
PostMI	-	72Hrs	-0.403	0.070	-5.747	46.951	< .00001

3.3 Estimated Marginal Means

Time

				95% Confidence Interval	
Time	Mean	SE	df	Lower	Upper
Baseline	2.750	0.053	49.276	2.643	2.857
PostMI	2.827	0.061	56.968	2.704	2.950
72Hrs	3.230	0.053	49.276	3.123	3.338
720Hrs	2.566	0.055	50.876	2.456	2.676

3.4 Effects Plots

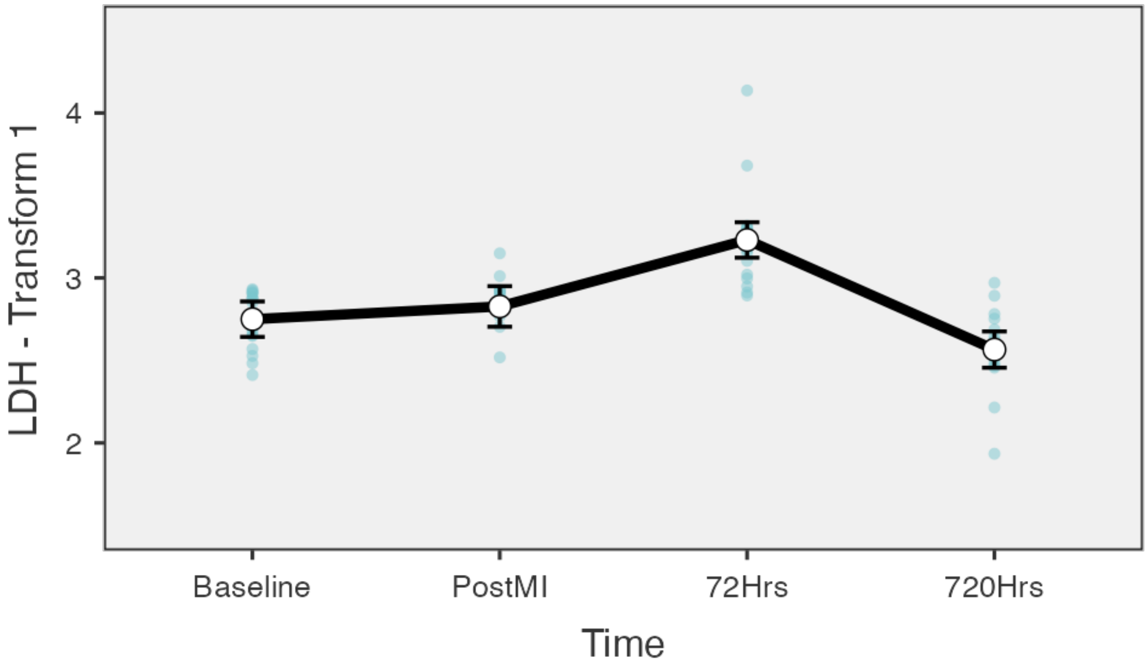

3.5 Assumption Checks

3.5.1 Q-Q Plot

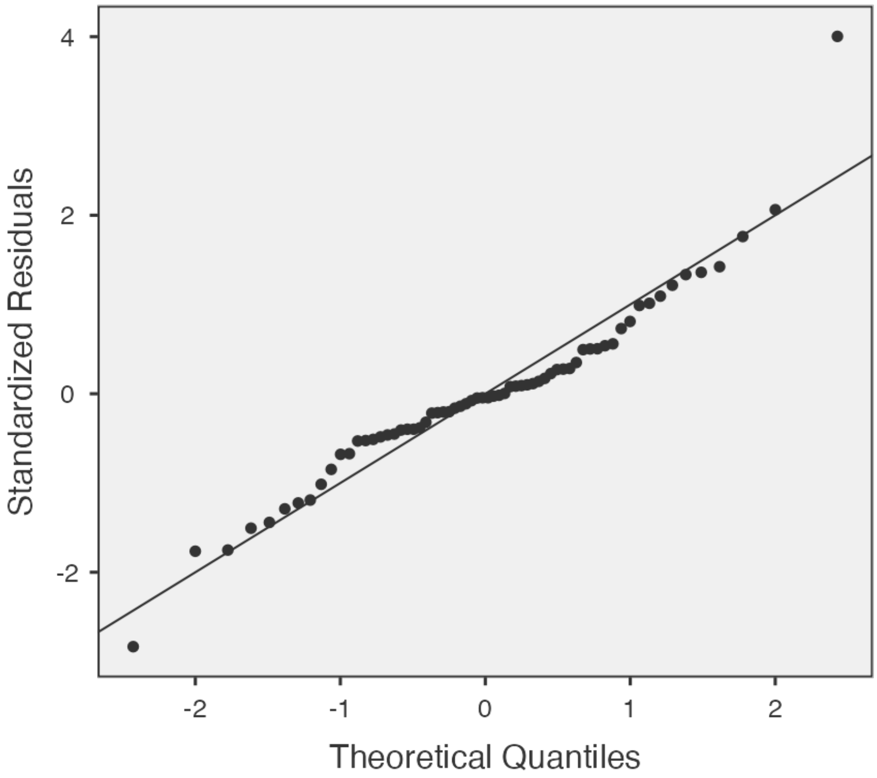

4 Mixed Model – CK level

Model Info

Info	
Estimate	Linear mixed model fit by REML
Call	CK - Transform 2 ~ 1 + Time+(1 | ID)
AIC	87.343
BIC	110.973
LogLikel.	-42.918
R-squared Marginal	0.442
R-squared Conditional	0.491
Converged	yes
Optimizer	bobyqa

4.1 Model Results

Fixed Effect Omnibus tests

	F	Num df	Den df	p
Time	18.706	3	46.539	< .00001

Note. Satterthwaite method for degrees of freedom

Fixed Effects Parameter Estimates

Names	Effect	Estimate	SE	95% Confidence Interval		df	t	p
				Lower	Upper			
(Intercept)	(Intercept)	3.132	0.061	3.011	3.255	16.672	51.149	< .00001
Time1	PostMI - Baseline	0.195	0.155	-0.112	0.497	48.055	1.255	0.21559
Time2	72Hrs - Baseline	0.400	0.141	0.106	0.671	44.797	2.830	0.00694
Time3	720Hrs - Baseline	-0.631	0.144	-0.883	-0.371	45.379	-4.398	0.00007

Random Components

Groups	Name	SD	Variance	ICC
ID	(Intercept)	0.131	0.017	0.087
Residual		0.424	0.180	

Note. Number of Obs: 66 , groups: ID 18

4.2 Post Hoc Tests

Post Hoc Comparisons - Time

Comparison							
Time		Time	Difference	SE	t	df	p_{holm}
72Hrs	-	720Hrs	1.031	0.144	7.178	45.743	< .00001
Baseline	-	720Hrs	0.631	0.144	4.394	45.743	0.00026

Post Hoc Comparisons - Time

Comparison							
Time		Time	Difference	SE	t	df	p_{holm}
Baseline	-	72Hrs	-0.400	0.141	-2.830	45.170	0.02076
Baseline	-	PostMI	-0.195	0.156	-1.249	48.378	0.38938
PostMI	-	720Hrs	0.826	0.158	5.217	49.184	0.00002
PostMI	-	72Hrs	-0.205	0.156	-1.315	48.378	0.38938

4.3 Estimated Marginal Means

Time

				95% Confidence Interval	
Time	Mean	SE	df	Lower	Upper
Baseline	3.141	0.105	60.707	2.932	3.350
PostMI	3.336	0.123	61.733	3.091	3.581
72Hrs	3.541	0.105	60.707	3.332	3.750
720Hrs	2.510	0.108	60.970	2.295	2.725

4.4 Effects Plots

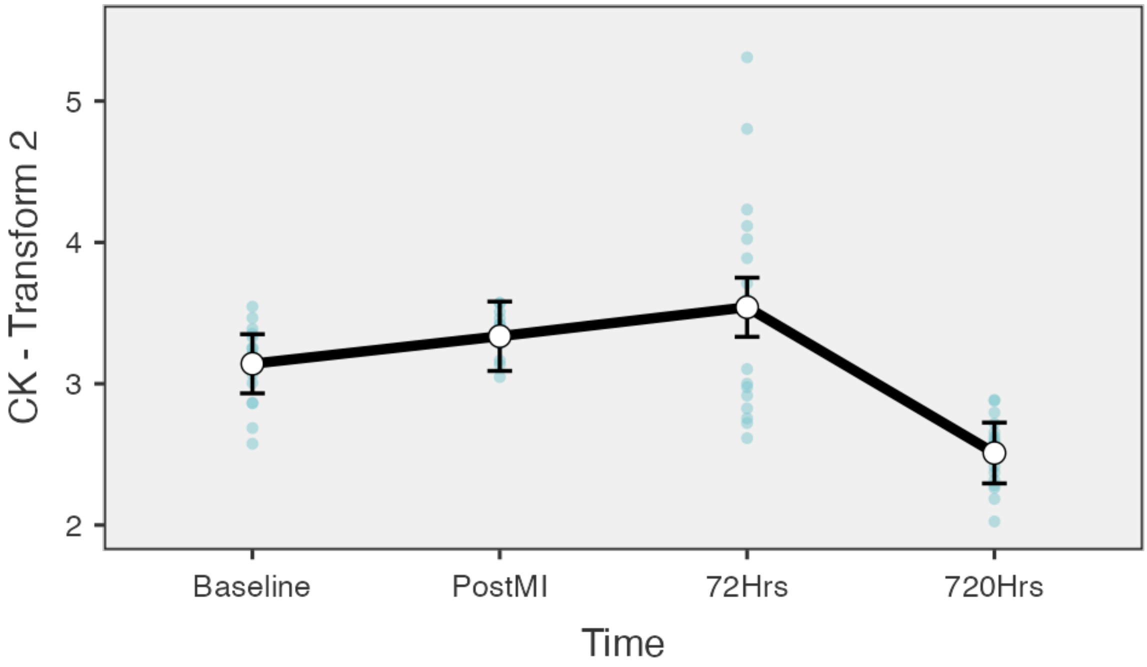

4.5 Assumption Checks

4.5.1 Q-Q Plot

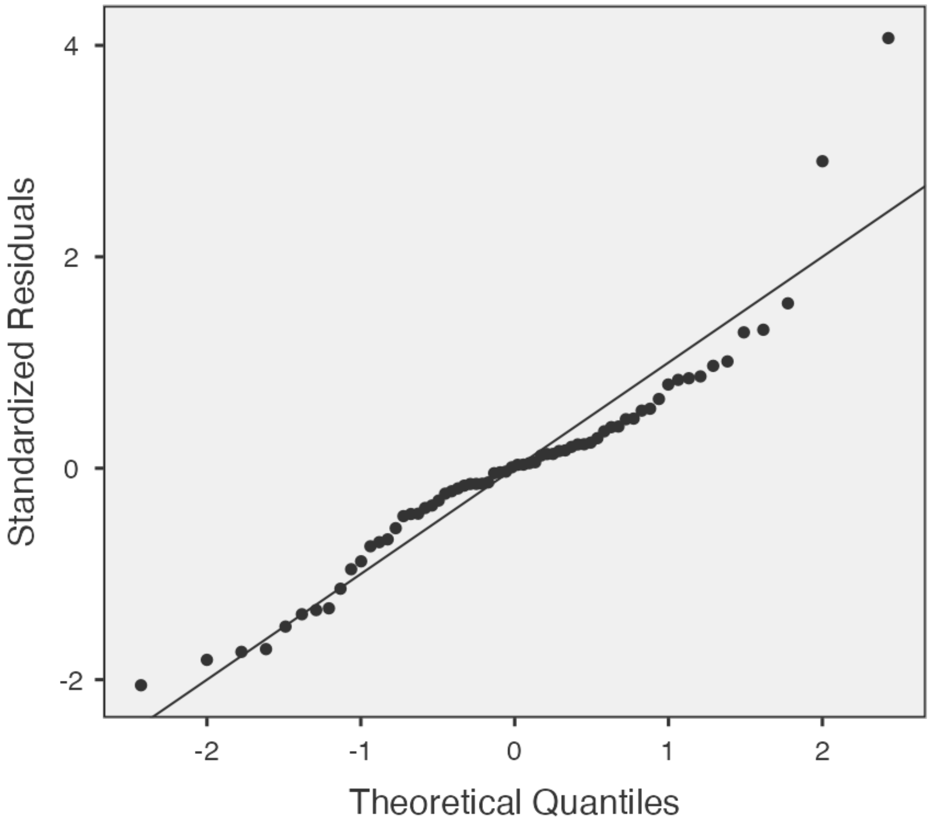

5 Mixed Model – Troponin level

Model Info

Info	
Estimate	Linear mixed model fit by REML
Call	Troponin - Transform 3 ~ 1 + Time+(1 | ID)
AIC	119.517
BIC	141.194
LogLikel.	-58.028
R-squared Marginal	0.795
R-squared Conditional	0.795
Converged	yes
Optimizer	bobyqa

Note. (Almost) singular fit. Maybe random coefficients variances are too small or correlations among them too large.

Note. boundary (singular) fit: see ?isSingular

5.1 Model Results

Fixed Effect Omnibus tests

	F	Num df	Den df	p
Time	83.904	3	62.000	< .00001

Note. Satterthwaite method for degrees of freedom

Fixed Effects Parameter Estimates

Names	Effect	Estimate	SE	95% Confidence Interval		df	t	p
				Lower	Upper			
(Intercept)	(Intercept)	1.456	0.070	1.322	1.588	62.000	20.786	< .00001
Time1	PostMI - Baseline	1.305	0.205	0.912	1.712	62.000	6.361	< .00001
Time2	72Hrs - Baseline	2.471	0.188	2.095	2.849	62.000	13.150	< .00001
Time3	720Hrs - Baseline	-0.153	0.191	-0.524	0.198	62.000	-0.804	0.42443

Random Components

Groups	Name	SD	Variance	ICC
ID	(Intercept)	0.000	0.000	0.000
Residual		0.564	0.318	

Note. Number of Obs: 66 , groups: ID 18

5.2 Post Hoc Tests

Post Hoc Comparisons - Time

Comparison			Difference	SE	t	df	p_{holm}
Time		Time					
72Hrs	-	720Hrs	2.624	0.191	13.751	45.988	< .00001
Baseline	-	720Hrs	0.153	0.191	0.803	45.988	0.42596

Post Hoc Comparisons - Time

Comparison							
Time		Time	Difference	SE	t	df	p_{holm}
Baseline	-	72Hrs	-2.471	0.188	-13.150	45.290	< .00001
Baseline	-	PostMI	-1.305	0.206	-6.325	49.225	< .00001
PostMI	-	720Hrs	1.459	0.209	6.974	50.157	< .00001
PostMI	-	72Hrs	-1.166	0.206	-5.649	49.225	< .00001

5.3 Estimated Marginal Means

Time

				95% Confidence Interval	
Time	Mean	SE	df	Lower	Upper
Baseline	0.550	0.133	62.000	0.284	0.815
PostMI	1.855	0.156	62.000	1.542	2.168
72Hrs	3.021	0.133	62.000	2.755	3.286
720Hrs	0.396	0.137	62.000	0.123	0.670

5.4 Effects Plots

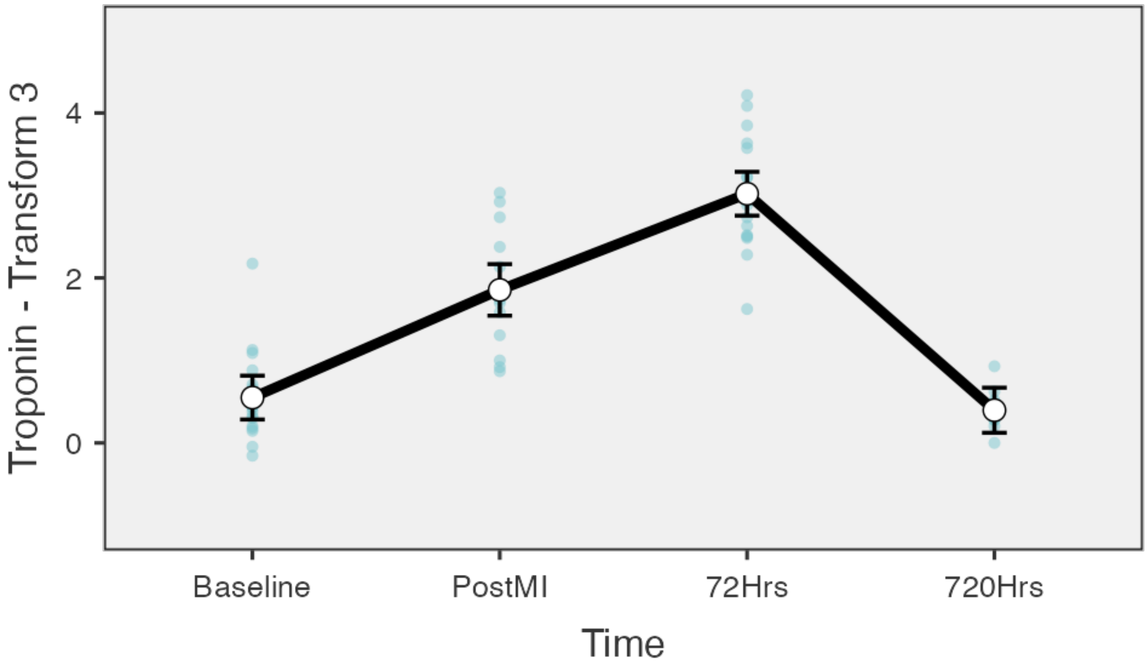

5.5 Assumption Checks

5.5.1 Q-Q Plot

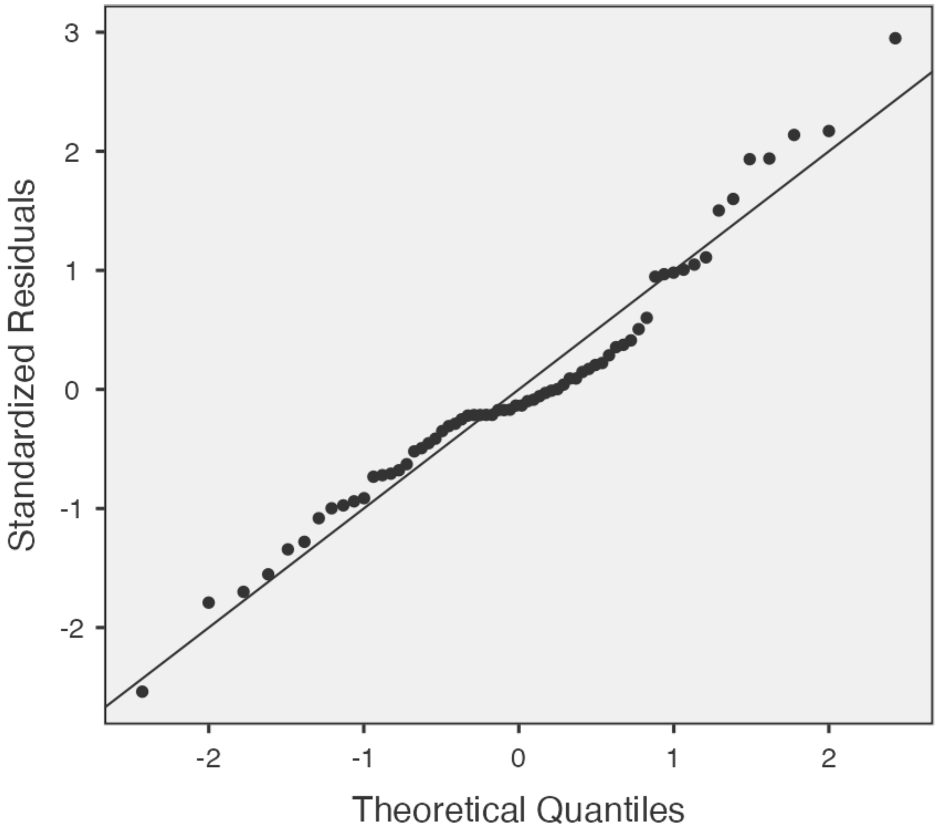

6 Repeated Measures ANOVA – AST/ALT ratio ("De Ritis)

Within Subjects Effects

	Sum of Squares	df	Mean Square	F	p	η^2_G
Timing	5.397	3	1.799	7.344	0.00067	0.269
Residual	8.084	33	0.245			

Note. Type 3 Sums of Squares

Between Subjects Effects

	Sum of Squares	df	Mean Square	F	p	η^2_G
Residual	6.565	11	0.597			

Note. Type 3 Sums of Squares

6.1 Post Hoc Tests

Post Hoc Comparisons - Timing

Comparison							
Timing		Timing	Mean Difference	SE	df	t	p
Baseline	-	Post	-0.522	0.265	11.000	-1.969	0.07469
	-	72 Hrs	0.067	0.156	11.000	0.428	0.67725
	-	30d FU	0.417	0.120	11.000	3.481	0.00514
Post	-	72 Hrs	0.588	0.243	11.000	2.423	0.03380
	-	30d FU	0.938	0.258	11.000	3.642	0.00387
72 Hrs	-	30d FU	0.350	0.104	11.000	3.372	0.00623
